# Supplementary material for: Regulation of Endoplasmic Reticulum–Mitochondria Tethering and Ca2+ Fluxes by TDP-43 via GSK3β
Source: Int J Mol Sci. 2021 Nov 1;22(21):11853. doi: 10.3390/ijms222111853 (PMC8584823; doi:10.3390/ijms222111853)
Supplement: Supplementary file 1 [file ijms-22-11853-s001.zip › ijms-1437540-supplementary.pdf]

Supplementary Figures and Tables

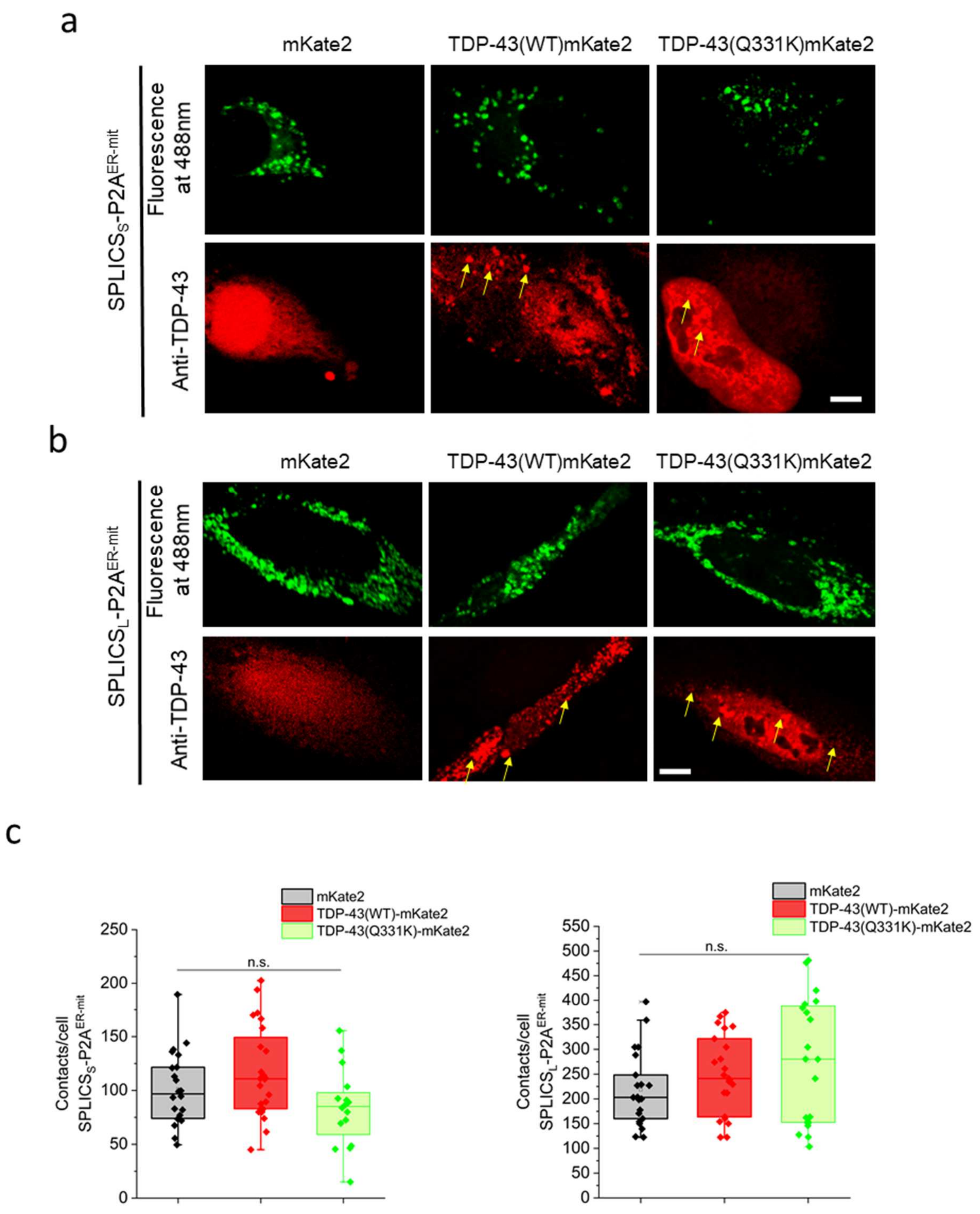

Supplementary Figure S1 – Effects of the transient overexpression of WT or the ALS-related Q331K mutated TDP-43 on mitochondria-ER contacts (MERCs) in HeLa cells. (a-b) Representative confocal micrographs of HeLa cells expressing the SPLICS$_S$ (a) or SPLICS$_L$ (b) probes (green signals; λ_{ex}, 488 nm) and co-transfected with plasmids coding for the mKate2 chimeric constructs (red signals; λ_{ex}, 594 nm) of TDP-

43, either WT (middle panels) or bearing the ALS-related Q331K missense mutation (right panels). As a control, cells were co-transfected with plasmid coding for mKate2 alone (left panels). Micrographs are representative of 3 biological replicates. Yellow arrows indicate TDP-43 inclusions. Scale bars, 5 µm. (c) Box plots report the quantification of $SPLICS_S$ (left panel) or $SPLICS_L$ (right panel) contacts per cell obtained after 3D rendering of complete z-stacks, other details are as in Figure 1. For $SPLICS_S$, n = 22, 24, 16 cells for mKate2, TDP-43(WT)-mKate2 and TDP-43(Q331K)-mKate2, respectively; for $SPLICS_L$, n = 21, 23, 20 for mKate2, TDP-43(WT)-mKate2 and TDP-43(Q331K)-mKate2, respectively; n.s., not significant, Kruskal-Wallis H test.

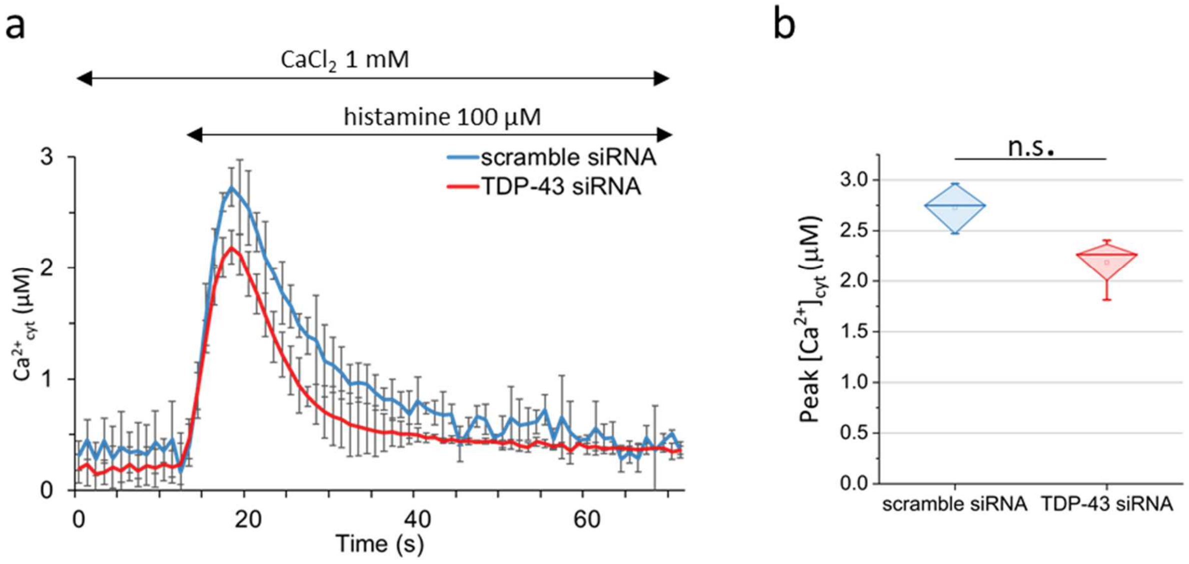

Supplementary Figure S2 – TDP-43 silencing does not significantly alter Ca^{2+} movements in the bulk cytosol after stimulation of $InsP_3$-induced Ca^{2+} mobilization from the ER. (a) Average kinetics of cytosolic [Ca^{2+}] transients after histamine (100 µM) addition at the indicated time point, monitored with the genetically targeted AEQcyt probe in HeLa cells transfected with TDP-43 or scramble siRNA oligonucleotides. Values are expressed as mean ± SD. (b) The diamond box plot reports the corresponding [Ca^{2+}] peaks. n=6 for both TPD-43 and scramble siRNA-treated cells. n.s., non-significant (Mann Whitney U test).

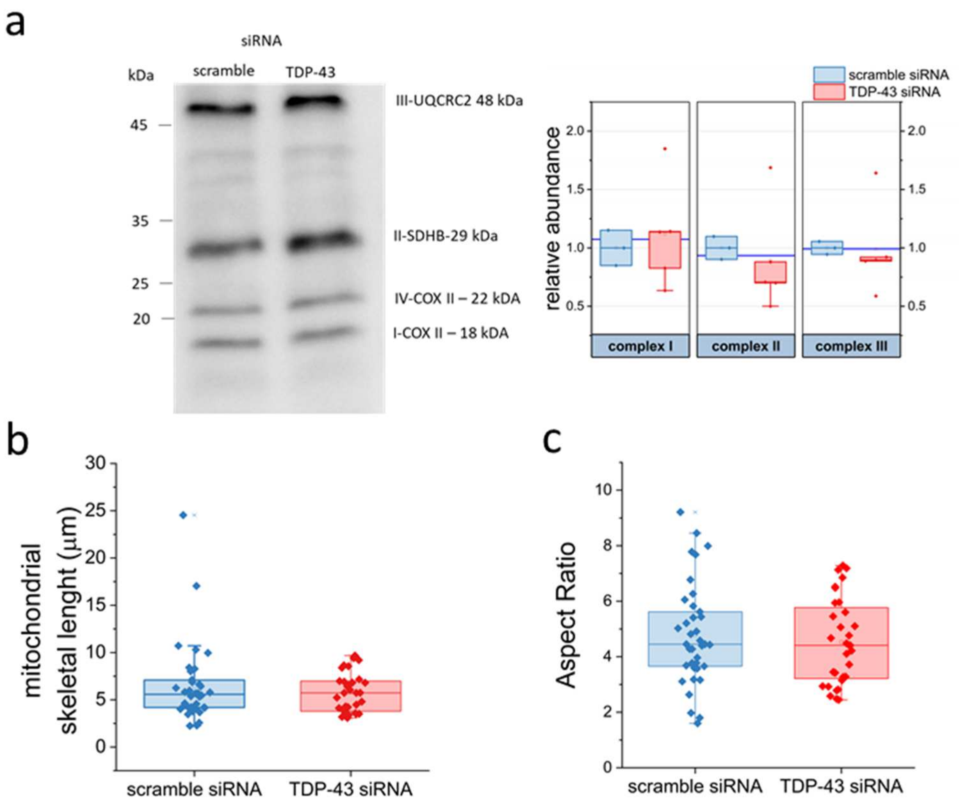

Supplementary Figure S3 – TDP-43 silencing does not significantly alter the expression of mitochondrial respiratory chain subunits or parameters of mitochondrial morphology. (a) Western blot (WB) analysis of mitochondrial oxidative phosphorylation (OXPHOS) complexes. An antibody cocktail against proteins representing four OXPHOS complexes was used to examine the expression of mitochondrial proteins in HeLa cells treated with scramble or TDP-43 siRNA oligonucleotides. A representative WB (left panel) and the densitometric analyses (right panel) are reported. n=4, (p-value > 0.5, Mann Whitney U test). (b-c) To evaluate mitochondrial parameters in Hela cells treated with scramble or TDP-43 siRNA oligonucleotides, a staining with an anti-TOM20 antibody was performed, and the 60 images of a 0.2 µm *z*-stack were analyzed using the Volocity 6.3 software. The mitochondrial skeletal length (b) and the aspect ratio (c) were determined as described in Materials and Methods. n=3 (p-value > 0.2, Mann Whitney U test).

Supplementary Table S1 – List of primers used to evaluate gene expression. Amplification efficiency was calculated according to the formula $10\hat{}(-\frac{1}{m})$ where m is the angular coefficient of the linear regression curve describing Ct in relationship to the known concentration of cDNA (serial dilutions were done).

Primer name	Sequence	Amplification efficiency
Hs-RMDN3-(PTPIP51)-For	CCAAAGCAGGAAGGGTATATA	108%
Hs-RMDN3-(PTPIP51)-Rev	GCCAACTTCATCCACCAT	
Hs-VAPB-For	CTCCGCCGCTAAGGAACAT	97%
Hs-VAPB-Rev	ACACACATTTCGGTCTGTCG	
Hs-GSK3A-For	ATCATCAAGGTGCTGGGAAC	105%
Hs-GSK3A-Rev	CCGTGTAGTTGGGGTTCATC	
Hs-GSK3B-For	CGCAGAACCTCTTGTTGGAT	107%
Hs-GSK3B-Rev	CGAAACATTGGGTTCTCCTC	
Hs-ITPR3-For	CGACATGCTTCATCTGTGGT	104%
Hs-ITPR3-Rev	TGCTCCAGCTTGATGTGTTC	
Hs-VDAC1-For	ACGTGGACTGAAGCTGACCT	108%
Hs-VDAC1-Rev	GTTAATGTGCTCCCGCTTGT	
Hs-HSPA9-For	GGCTGGAGACAACAAACTCC	96%
Hs-HSPA9-Rev	CTCACGTCCTGTGCCTTTATC	
Hs- HSPA5-For	CTGGTGTGCTCTCTGGTGAT	91%
Hs-HSPA5-Rev	CTTCTTGGTAGGCACCACTG	
Hs-MCU-For	AAAGGAGCCAAAAAGTCACG	101%
Hs-MCU-Rev	TGCAGATCAATCTTTTTCACCA	
Hs-MICU1-For	AGACCACGTGTGTGATGTGG	99%
Hs-MICU1-Rev	TATTGCTCAGTTCGCCATTG	
Hs-TPM2-For	TGGCAAAGTTGGAGAAAACC	94%
Hs-TPM2-Rev	TGGTCCAAGGTCTGGTGAAT	
Hs-MICU2-For	GTTGTCAGCAGGAGAGAGCA	113%
Hs-MICU2-Rev	TGCATGGCAATAGCAAAGTC	
Hs-MFN1-For	TACCACTTTTGCTCGCCTGT	103%
Hs-MFN1-Rev	TCATGGTCACCAAAGCAATC	
Hs-MFN2-For	TCTGGGACCTTTGCTCATCT	100%
Hs-MFN2-Rev	ACGAGACTGGGTGCTTCATT	
